# Incidence and risk factors for influenza-like-illness in the UK: online surveillance using Flusurvey

**DOI:** 10.1186/1471-2334-14-232

**Published:** 2014-05-01

**Authors:** Alma J Adler, Ken TD Eames, Sebastian Funk, W John Edmunds

**Affiliations:** 1 Department of Infectious Disease Epidemiology, London School of Hygiene & Tropical Medicine, London, UK

**Keywords:** Influenza, Online surveillance, Risk factors

## Abstract

**Background:**

Influenza and Influenza-like-illness (ILI) represents a substantial public health problem, but it is difficult to measure the overall burden as many cases do not access health care. Community cohorts have the advantage of not requiring individuals to present at hospitals and surgeries and therefore can potentially monitor a wider variety of cases. This study reports on the incidence and risk factors for ILI in the UK as measured using Flusurvey, an internet-based open community cohort.

**Methods:**

Upon initial online registration participants were asked background characteristics, and every week were asked to complete a symptoms survey. We compared the representativeness of our sample to the overall population. We used two case definitions of ILI, which differed in whether fever/chills was essential. We calculated ILI incidence week by week throughout the season, and investigated risk factors associated with ever reporting ILI over the course of the season. Risk factor analysis was conducted using binomial regression.

**Results:**

5943 participants joined the survey, and 4532 completed the symptoms survey at least twice. Participants who filled in symptoms surveys at least twice filled in a median of nine symptoms surveys over the course of the study. 46.1% of participants reported at least one episode of ILI, and 6.0% of all reports were positive for ILI. Females had slightly higher incidence, and individuals over 65 had the lowest incidence. Incidence peaked just before Christmas and declined dramatically during school holidays. Multivariate regression showed that, for both definitions of ILI considered, being female, unvaccinated, having underlying health issues, having contact with children, being aged between 35 and 64, and being a smoker were associated with the highest risk of reporting an ILI. The use of public transport was not associated with an increased risk of ILI.

**Conclusions:**

Our results show that internet based surveillance can be used to measure ILI and understand risk factors. Vaccination is shown to be linked to a reduced risk of reporting ILI. Taking public transport does not increase the risk of reporting ILI. Flusurvey and other participatory surveillance techniques can be used to provide reliable information to policy makers in nearly real-time.

## Background

Each year influenza causes a substantial burden of illness. Even in a non-pandemic year, influenza is estimated to cause between 250,000 and 500,000 deaths worldwide [1]. In some years, the burden can be much higher. Serological studies estimated that in the second wave of the 2009 pandemic, in the United Kingdom (UK) 49% of under five year olds, 59% of 5-14 year olds, 35% of 15-24 year olds, and 25% of 25-44 year olds were infected [2]. While many cases of influenza can be mild, serious complications can occur, particularly in the very young, very old, and individuals with pre-existing health conditions [1]. In order to help reduce the impact of influenza, it is important to understand who is most likely to be infected. Despite the high burden of influenza there is little quantitative information about risk factors that may inform potential preventive strategies.

The incidence of influenza is not generally recorded by public health surveillance, since it is impossible to conclusively know if an individual has influenza without virological confirmation, which is very rarely performed. As a result, most reports are of cases of influenza like illness, or ILI.

Traditional ILI monitoring relies on general practitioner (GP) and hospital reports. These methods, although useful, have the main limitation that they require individuals to seek health care [3]. Given that individuals with ILI who seek health care are not a random sample of cases, but are likely to differ in a number of characteristics (for example gender, socio-economic class, severity of illness), there is a high likelihood of selection bias. Additionally, during pandemics or other periods of high incidence, uncomplicated cases may be discouraged from presenting to health care, which would further distort the picture emerging from routine (health-care dependent) surveillance [4]. Finally it is difficult to link reports of ILI to behavioural and biological risk factors, since much of this information will be absent from GP records. As a result estimates of the incidence of influenza and of associated risk factors based on routine surveillance may be unreliable and unrepresentative.

An alternative is to measure the incidence and risk-factors via community cohort surveys. The internet allows these to be conducted at relatively low cost and in a way that is convenient for participants. Hence this method has become increasingly used across Europe [5-7] and elsewhere [8,9], to estimate the incidence of ILI, and to understand the epidemiology of ILI and the effectiveness of control efforts [10]. Each week, participants are asked to record the presence or absence of symptoms, by which means both a numerator and denominator are obtained. Background information collected when participants register allows real-time estimates of regional incidence by age group and assessment of the role of other risk factors.

The UK web-based surveillance “Flusurvey” (https://flusurvey.org.uk/) was launched in July 2009 during the H1N1 pandemic [11], and has now run for five years. Flusurvey is linked to Influenzanet (https://www.influenzanet.eu/) a Europe-wide ILI monitoring system, currently running in nine countries. Here we present UK specific incidence estimates and risk factor analyses for ILI from the 2012-2013 season.

## Methods

Participants include any resident of the UK recruited into Flusurvey between November 22^nd^ 2012 and April 14^th^ 2013. Flusurvey was approved by the London School of Hygiene & Tropical Medicine Ethics Committee (Application number 5530).

### Structure of the survey

Upon initial registration, participants were asked a set of background questions about their age, gender, socio-economic status, household composition, geographical location, vaccine status, use of public transport, employment, and educational status. Participants were also asked questions about pre-existing health-conditions including use of medication for diabetes, asthma and other respiratory diseases, heart disease, kidney disorders, and immune-compromising conditions. Further details about the background questions are available upon request. In addition to being able to register themselves, participants were able to register on behalf of others (e.g. members of their household). Participants who had registered for Flusurvey in previous years were required to fill in their background information again to ensure it was up to date. During the course of the season we reminded participants in the weekly email to update their vaccination status if necessary

Each week a reminder email was sent asking participants to complete a symptoms survey, whether or not they had any symptoms. Participants were asked to indicate on a list of symptoms which, if any, they had experienced in the past week or since their last symptoms survey report. Participants who reported any symptoms were asked a set of follow-up questions, detailing onset date, suddenness of onset, and health seeking behaviours. The full list of symptoms is found in Table 1.

**Table 1:** List of symptoms in the survey

No symptoms	Chest pain
Fever	Feeling tired or exhausted
Chills	Loss of appetite
Runny/Blocked nose	Coloured sputum/phlegm
Sneezing	Watery, bloodshot eyes
Sore throat	Nausea
Cough	Vomiting
Shortness of breath	Diarrhoea
Headache	Stomach Ache
Muscle/Joint Pain	Other

### Demographics

Our sample was compared to the UK population to assess its representativeness. Background demographic data for the UK was taken from the office of National Statistics website [12]. Representativeness with respect to vaccination uptake was assessed using influenza vaccine uptake reports from the Public Health England (PHE) Website [13].

### Case definitions

A diagnosis of influenza-like-illness (ILI) was made based on participants’ self-reported symptoms. Since there is no case definition of ILI upon which everyone agrees, and recognising the possible sensitivity of any results to the precise definition chosen, in this study we consider two different definitions of ILI. First, ILI^ECDC^, using European Centre for Disease Prevention and Control (ECDC) definition which required: Sudden onset of symptoms; at least one of fever or chills, malaise, headache, muscle pain; at least one of cough, sore throat, shortness of breath. In the Additional file 1: Appendix we consider a stricter ILI definition which was the ECDC definition but additionally required a fever.

### Data management

Participants received a reminder each week to complete their symptoms survey. However, not all participants responded on the day the reminder was sent, so completed surveys are not evenly spread. On less than six percent of occasions participants submitted multiple symptoms surveys on the same day. Multiple responses by individuals on the same day were treated as follows: if there was one symptom surveys that included a report of ILI^ECDC^ that one was kept, and if not, the most recent one was kept. If an individual reported symptoms in consecutive surveys, they were asked whether the symptoms belonged to the same episode of illness. If symptoms were reported to be from the same episode, they were only included as an incident case once. As it was believed that individuals were more likely to register for Flusurvey when they had an ILI, upon enrolment, the first symptoms survey submitted by each participant was dropped when estimating weekly incidence. For risk factor analyses, only individuals who had submitted at least two symptoms surveys were included in the sample.

### Statistical analysis

#### Measure of disease frequency

Data were analysed using R-studio version 0.97.237 and R 2.15.2. Unadjusted incidence was calculated both as a participant’s risk of reporting ILI at least once in a season, as well as a risk per symptoms survey. The overall risk was calculated by total number of positive reports over total number of reports. Weekly ILI^ECDC^ incidence by age group was plotted alongside actual recorded percent positive confirmed influenza swabs collected by PHE’s Respiratory DataMart system [14].

### Risk factor analysis

For risk factor analysis, we considered factors potentially related to the chance of an individual reporting ILI at least once during the season. Risk factors included geographical area, vaccine status, pre-existing health conditions, employment, use of public transportation, household makeup, exposure to children, gender and age group.

Data were analysed using a generalised linear model using a binomial regression; odds ratios (and 95% confidence intervals) were reported as measures of association.

The odds of reporting ILI at least once were adjusted by the number of reports an individual submitted; age group (under 18, 18-24, 25-34, 35-44, 45-64, 65+); gender; influenza vaccination status; region (13 NHS regions); employment status (studying, working full or part time, homemaker, unemployed); whether the participant was in contact with groups of 10 or more children (children defined as under the age of 18) in the course of a typical day; whether the participant lived with children; pet ownership; having pre-existing health conditions (asthma, diabetes, kidney disease, heart disease, immuno-compromising conditions); allergies; smoking (any number of cigarettes); regular use of public transport. Because some of the covariates such as smoking and interacting with a child may have differences in effect in participants under the age of 18, a sensitivity analysis was conducted removing children from the analysis.

## Results

### Demographics

5943 participants were recruited. Of those 1511 only completed the symptoms survey once, leaving 4532 participants that were included in the analysis. Participants’ characteristics are shown in Tables 2 and 3. Participants included in the analysis completed a median of nine symptoms surveys and a mean of 9.9 symptoms surveys.

**Table 2:** Characteristics of all participants, and participants with at least two reports included in the study

**All registered participants**		**Participants with 2 or more symptoms surveys**					
**Age group**	**Participants**	**Participants (% of all participants)**	**Percent of Flusurvey sample**	**Percent of UK population**	**Female n (%)**	**Risk factor n (%)**	**Vaccine n (%)**
0-17	403	274 (68.0)	6.2	21.0	136 (49.6)	30 (10.9)	24 (8.8)
18-24	382	202 (52.9)	4.6	9.4	142 (70.3)	30 (14.9)	25 (12.4)
25-34	1132	774 (68.4)	17.5	13.6	557 (72.0)	100 (12.9)	153 (19.8)
35-44	1281	903 (70.5)	20.4	13.5	611 (67.7)	138 (15.3)	204 ( 22.6)
45-64	2138	1778 (83.2)	40.1	25.4	1150 (64.7)	326 (18.3)	583 (32.8)
65+	607	501 (82.5)	11.3	17.1	288 (57.5)	183 (36.5)	450 (89.8)
% of total included in our study					75.9%	79.1%	84.4%

**Table 3:** Proportion of participants by region and risk factors

	**Number participants**	**% of Flusurvey sample**	**% of total in the UK**
Region			
East Midlands	260	5.8	8.7
East of England	470	10.5	8.9
London	887	19.8	12.6
North East	102	2.3	4.1
North west	366	8.2	11.1
Northern Ireland	64	1.4	2.9
Scotland	305	6.8	8.5
South Central	440	9.8	6.4
South East Coast	351	7.8	6.8
South West	422	9.4	8.2
Wales	201	4.5	5.0
West Midlands	299	6.7	8.7
Yorkshire & Humberside	310	6.9	8.2
**Other risk factors**			
Smoker	444	9.8	21
Has a pet	1974	43.6	n/a
Takes public transport	1456	32.1	n/a
In contact with children	833	18.4	n/a
Has children	1538	33.9	n/a

Females were over-represented in our sample (64% female). Children were under-represented (6.2% of our sample versus 21% of the UK population), and individuals aged 35-64 were over-represented. About ten percent of all participants (456) were registered by proxy however due to the anonymous nature of our survey we do not know about how many individuals participated from the same household, for this reason we did not cluster by household.

There were regional differences in the sample, with London being over-represented and Northern Ireland being the least well represented region in the survey (Table 3).

Participants had a higher rate of vaccination than the UK population (Table 4), suggesting that Flusurvey participants may have more awareness of influenza than the UK population in general. Table 5 shows number of reports submitted per week.

**Table 4:** Vaccination characteristics of participants with at least two reports compared to overall UK population

**Risk group**	**% of Flusurvey sample vaccinated 2012-2013 season**	**% of total in the UK vaccinated 2012-2013 season**
Over 65	89.8	73.4
Pregnant women	65.0	40.3
Underlying health condition	70.3	51.3
Healthcare workers	57.2	45.6
Total	32.2	n/a

**Table 5:** Number of submitted reports by week

**Week ending:**	**Number reports:**
25-Nov	2496
02-Dec	2253
09-Dec	2624
16-Dec	2552
23-Dec	2748
30-Dec	2379
06-Jan	3065
13-Jan	2982
20-Jan	2810
27-Jan	2715
03-Feb	2766
10-Feb	2675
17-Feb	2641
24-Feb	2551
03-Mar	2519
10-Mar	2457
17-Mar	2400
24-Mar	2386
31-Mar	2102
07-Apr	2350
14-Apr	2267

### Unadjusted ILI incidence

46.1% (95% CI 44.6, 47.5) of individuals reported at least one episode of ILI^ECDC^ during the season, and 6.0% (95% CI 5.7, 6.2) of symptom reports were positive for ILI^ECDC^. Incidence was higher in females, with 49.6% (95% CI 47.8, 51.5) of females reporting at least one ILI^ECDC^ compared to 39.7% (95% CI 37.3, 42.2) of males. 6.9% (95% CI 6.6, 7.2) of symptoms surveys submitted by females were positive for ILI^ECDC^, compared to 4.6% (95% CI 4.3, 4.9) of surveys submitted by males. Participants over 65 had the lowest positive proportion of total reports: 33% (95% CI 29.1, 37.0) of individuals had at least one episode of ILI^ECDC^ and 2.9% (95% CI 2.5, 3.2) of all surveys were positive; participants between 45 and 65 had the highest proportion of having at least one report of ILI^ECDC^ (50.1%, 95% CI 47.8, 52.5); participants under the age of 18 had the highest proportion (7.9%, 95% CI 6.8, 9.1) of positive symptoms surveys (Table 6). Analysis of ILI as measured using the stricter ILI^fever^ found similar patterns (see Additional file 1: Appendix 1).

**Table 6:** Overall and weekly incidence of ILI using ECDC and fever definitions

**Group**	**Overall incidence (at least once)**	**95% CI**		**Incidence per report**	**95% CI**	
0-17	48.9	42.8	55.0	7.9	6.8	9.1
18-24	41.1	34.2	48.2	6.8	5.6	8.2
25-34	44.4	40.9	48.0	6.4	5.8	7.0
35-44	48.2	44.9	51.5	7.4	6.8	8.0
45-64	50.1	47.8	52.5	6.3	5.9	6.6
65+	33	29.1	37.0	2.9	2.5	3.2
Male	39.7	37.3	42.2	4.6	4.3	4.9
Female	49.6	47.8	51.5	6.9	6.6	7.2
East Midlands	45.4	39.2	51.7	5.6	4.8	6.6
East England	44.9	40.3	49.5	5.8	5.1	6.5
London	42.3	39.0	45.6	5.1	4.7	5.5
North East	45.1	35.2	49.3	6.8	5.3	8.7
North West	46.2	41.0	51.4	6.5	5.7	7.4
Northern Ireland	62.5	49.5	74.3	9.7	7.3	12.6
Scotland	44.9	39.2	50.7	5.9	5.1	6.8
South Central	48.9	44.1	53.6	6.0	5.3	6.7
South East Coast	47.9	42.5	53.2	6.5	5.7	7.4
South West	49.5	44.7	54.4	6.8	6.0	7.6
Wales	43.8	36.8	50.9	6.4	5.3	7.6
West Midlands	44.5	38.8	50.3	5.2	4.4	6.0
Yorkshire & Humberside	50.3	44.6	56.0	6.6	5.8	7.6

**Figure 1:**
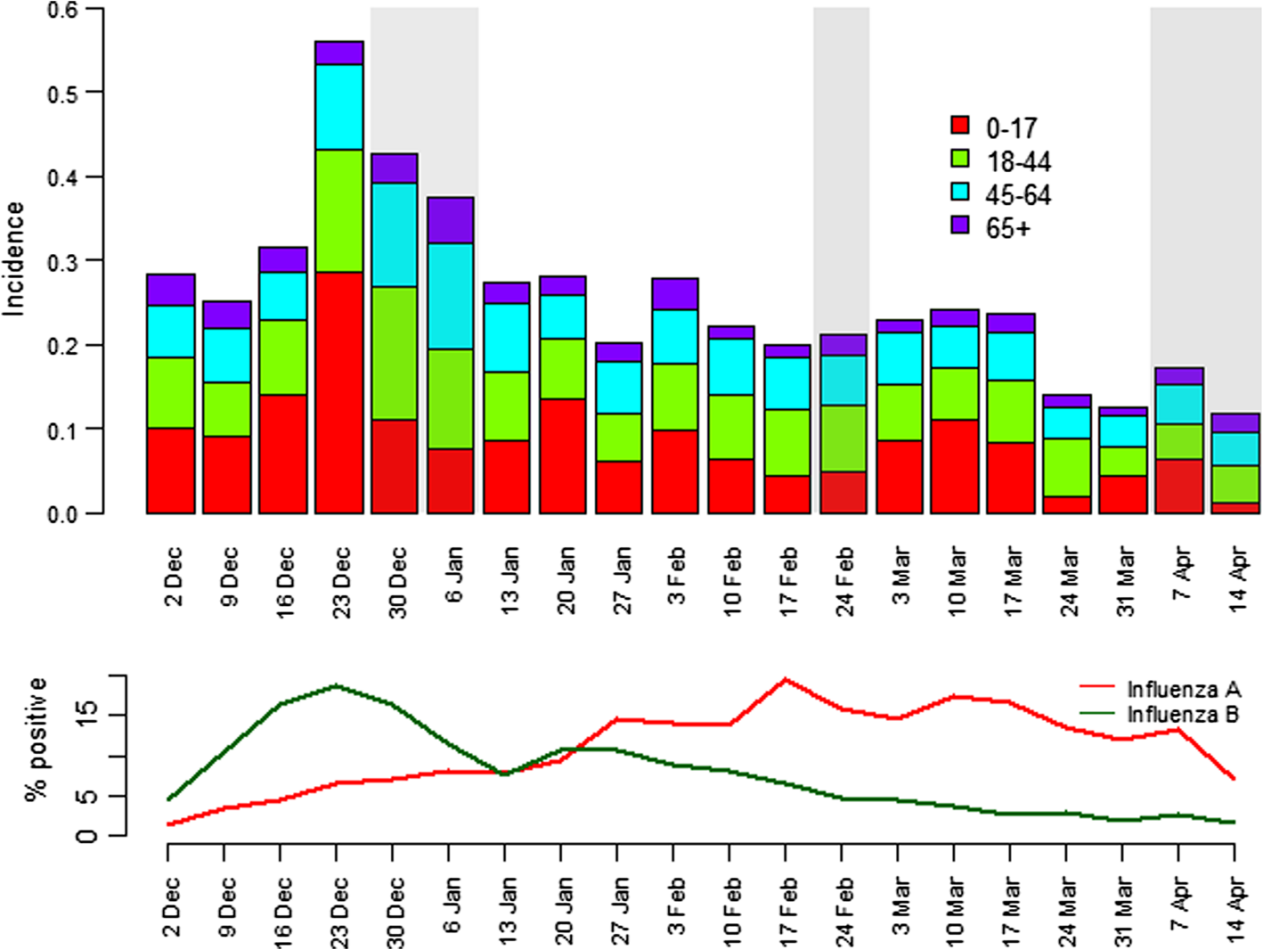
**Weekly incidence by age group.** Highlighted areas show school holidays (taken from http://www.halftermdates.co.uk/ and may not be representative of entire population). The lower panel shows the proportion of samples testing positive for Influenza A and Influenza B. The Respiratory DataMart System is a laboratory surveillance tool to monitor influenza and other respiratory viruses based on collated lab results from a network of Public Health England (PHE) and NHS laboratories in England. Respiratory swabs from primary and secondary care are tested for a variety of viruses using real time polymerase chain reaction (RT-PCR) assays. Weekly results are published in the PHE National Influenza Report (http://www.hpa.org.uk/Topics/InfectiousDiseases/InfectionsAZ/SeasonalInfluenza/EpidemiologicalData/03influsweeklyreportpdfonly/) First week of reporting is not included as responses may include all cases of ILI since the previous season.

The highest incidence of ILI in any age group came the week ending December 23, in children under the age of 18. In the following week, which coincided with school closures for the Christmas holidays, there was a substantial fall in cases in under 18 year olds, but a small rise in incidence in the older age groups. With the re-opening of schools, there was a corresponding increase in cases of ILI in the youngest age group. The older age groups, particularly the 19-45 year olds, had a similar pattern to the under 18 year olds, but slightly delayed. This pattern was repeated on a smaller scale when the schools broke up for Easter holidays, with the older age groups having a slightly delayed peak. Figure 1 shows weekly incidence by age group, with shaded areas representing school holidays, although we note that there is some local variation in holiday dates (http://www.halftermdates.co.uk/). The bottom portion of the graph gives the percent positivity of laboratory confirmed Influenza A and Influenza B reported by PHE’s Respiratory DataMart system [14]. The increase of ILI just before the Christmas holidays corresponds to an increase of Influenza B recorded by PHE [14]. The peak in ILI incidence later in the season corresponds to an increase in cases of Influenza A. The first week of reporting is not included on Figure 1 because first week reporting may be inflated due to participants reporting illness over a potentially longer time period.

### Risk factor analysis

Results of multivariate analysis for factors influencing the risk of reporting at least one episode of ILI^ECDC^ are found in Table 7. Working with groups of patients and the elderly was not found to have an effect on having ILI^ECDC^. Our findings suggest that not being vaccinated against influenza was associated with the highest odds of getting ILI^ECDC^ at least once (OR 1.94, 95% CI 1.66, 2.27). Having an underlying health condition such as asthma or diabetes also substantially increased participant’s odds of reporting at least one ILI^ECDC^ (OR 1.53, 95% CI 1.27, 1.83). Other factors showing an association included being female (OR 1.52, 95% CI 1.33, 1.73) having daily contact with large groups of children (OR 1.47, 95% CI 1.22, 1.77), smoking (OR 1.32, 95% CI 1.07, 1.64), and being aged between 35 and 64. (35-44 OR 1.62, 95% CI 1.11, 2.08; 45-64 OR 1.55, 95% CI 1.14, 2.10).

**Table 7:** Risk factors of having at least one ILI using ECDC definition

**Variable**	**OR**	**95% CI**		**P**
Female	1.52	1.33	1.73	0.03
Unvaccinated	1.94	1.66	2.27	<0.001
18-24	0.95	0.64	1.42	0.80
25-34	1.12	0.81	1.55	0.50
35-44	1.42	1.04	1.94	0.03
45-64	1.51	1.12	2.03	0.007
65+	0.95	0.65	1.38	0.79
Contact with children	1.47	1.22	1.77	<0.001
Live with children	1.10	0.95	1.28	0.305
Smoker	1.32	1.07	1. 64	0. 01
Take public transport	0.91	0.78	1.05	0.183
Underlying health condition	1.53	1.27	1.83	<0.001
Employment status	0.92	0.78	1.10	0.066

People who took public transport were at no greater odds of reporting ILI^ECDC^ than people who did not (OR 0.91, 95% CI 0.78, 1.05). Likewise, there was no statistically significant difference in the odds of reporting ILI^ECDC^ by region. Multivariate results using the stricter ILI^fever^ definition were similar, and can be found in Additional file 1: Appendix 2. Multivariate results removing children from the analysis were also similar and are found in Additional file 1: Appendix 3.

## Discussion

Our results show that online ILI surveillance can be an effective tool for measuring ILI incidence, and that volunteers are willing to take part in ILI surveillance. The analyses shown in this paper highlight the advantages of community surveillance of ILI compared to traditional GP surveillance techniques. Six percent of all symptom reports were positive for ILI in the 2012-2013 flu season based on a sample of 4523 participants. When standardised by age to the UK population, this corresponds to an incidence of 6.3 per 100 person weeks. As in previous years, we showed that incidence was lowest in participants over 65. This is in part because individuals over 65 were more likely to be vaccinated. There were no significant regional differences. Our results show that people with underlying health conditions were more likely to report ILI, a similar result to that found using telephone surveillance in the United States [15].

In our study, females were more likely to report ILI. This pattern was still evident when adjusting for living with children, or for daily contact with groups of children (which would include teachers and nursery workers). This same pattern was observed in a study of healthy adults in Australia [16]. We speculate that females may be more likely to take care of symptomatic individuals, and therefore be exposed to illness or that the different influenza rates in females may be due to physiological differences [17].

The highest rates of ILI were in the youngest age category, in agreement with other community-based findings [18]. The decline in incidence is coincident with Christmas holidays, and the resurgence of cases in this age group in the new year, after schools had reopened, is similar to other data [7,19-21]. The observation that incidence in the 19-45 year olds lagged behind the youngest age group, suggests that under 18s may have brought the illness back to their families.

Our results also show that taking public transport does not increase your risk of reporting an ILI regardless of definition used. Analyses from other European countries and over different seasons confirm this finding [Van Noort S, Codeço C, Kopperschaar C, Van Ranst M, Gomes M: Influenzanet: ILI trends, behaviour and risk factors in cohorts of internet volunteers, submitted, [22], though others have found the opposite [23].

Our results suggest that influenza vaccination gives some protection against ILI. With any definition of ILI considered, not being vaccinated was the greatest predictor of reporting an ILI. This result is consistent with results from previous years [10]. Some ILI was still reported in participants who reported vaccination. This can in part be attributed to the fact that the vaccine is only effective against influenza and we are measuring ILI some of which will not be due to influenza. Restricting the analysis of our data to the largest peak of virologically confirmed influenza (9^th^ of December through 30^th^ of December), the OR for being unvaccianted increased to 2.14 (95% CI 1.68, 2.74).

### Strengths and weaknesses of the study

The use of internet based surveillance has a number of limitations.

All of our data are based on self-reports of symptoms. As such we are unable to comment on influenza, only on ILI. However, most GP surveillance is also based on ILIs since most cases are not virologically confirmed. There is little information on the specificity and sensitivity of ILI during the influenza season. As shown by Figure 1, our measured incidence of ILI corresponded well to virological surveillance recorded by PHE. Ideally, weekly swabbing of Flusurvey participants would be used to assess the proportion of ILI cases that are confirmed influenza. Currently the costs of swabbing have not allowed this. However we would like to attempt this in the future.

Our sample does not represent a random sample; by nature of its design it is biased towards internet users, and people who are willing to fill in surveys. Additionally Flusurvey participants are more likely to be from London, be female, have risk factors and be vaccinated than the UK general population. Other methods such as telephone surveillance can be used to overcome these limitations [15,24], however these surveillance techniques are more expensive, time absorbing, have representativeness problems of their own, and still have the problem of self-reports of ILI.

Our decision to exclude first reports in incidence estimates may have caused us to underestimate the overall amount of ILI in our sample. When including first reports, 7.8% (95% CI 7.6, 8.0) of total symptom reports were positive. When removing first reports 6.0% (95% CI 5.7, 6.2) were positive. However, the survey asks participants if they had had any symptoms since the last time they logged in, so participants’ first reports may reflect symptoms that occurred outside the current flu season.

Our risk factor analysis is restricted to the questions that are asked at the beginning of the survey. Our multivariate analysis suggests that being under the age of 18 is not a risk factor for reporting ILI. However, being in contact with groups of children is. Similarly, living with children was not seen to be a risk factor, but this is colinear with being a child. As a result, due to the wording of this question, it is difficult to understand the implications of these results. If we had asked the question in another way, for example, are you an adult who works with children, we may have been better able to separate the effects of working with children versus being a child. It is plausible that living with children is a risk factor, but the colinearity with being a child masks this relationship. Sensitivity analysis showed no substantial difference in results when children were removed from the analysis.

Flusurvey does have many strengths. In effect the survey consists of an online cohort. We were able to attribute illness directly (although anonymously) to individuals, thus understanding their individual risk factors. This is in comparison to other online influenza tracking sights such as Flu Near You (https://flunearyou.org/) which is run in the United States. They do not ask background questions about individual risk factors, and can therefore only report on influenza prevalence. Flutracking (https://www.flutracking.net/), based in Australia, only asks about gender, age, and working with patients. Only Flusurvey and the other members of Influenzanet ask in-depth questions about risk factors. The data are available in real-time allowing rapid examination of these risk factors and the effectiveness of control programmes. Finally, the size of the cohort can be expanded at very little additional cost.

## Conclusion

Overall we found that 6.0 percent of all symptoms surveys were positive for ILI in the 2012-2013 flu season based on a sample of 4523 participants. Age standardised to the UK population this corresponds to an incidence of 6.3 per 100 person weeks. Females reported higher incidence of ILI than males, and the highest incidence was in under 18 year olds. This internet-based cohort has confirmed that failure to be vaccinated was the most important risk factor for ILI during the 2012-13 influenza season in the UK. It has also demonstrated that public transport use does not appear to be a risk-factor for ILI, whereas smoking and having a pre-existing health condition are. Such participatory surveillance systems have the ability to provide reliable information to policy-makers in close to real-time.

## Abbreviations

ECDC: European Centre for Disease Control; GP: General practitioner; ILI: Influenza-like-illness; PHE: Public Health England; UK: United Kingdom.

## Competing interests

The authors declare that they have no competing interests.

## Authors’ contributions

AJA-Data collection, data analysis, data interpretation, writing KTDE-Study design, data interpretation, writing SF-Data collection, data analysis WJE-Study design, data interpretation, writing. All authors read and approved the final manuscript.

## Pre-publication history

The pre-publication history for this paper can be accessed here:

http://www.biomedcentral.com/1471-2334/14/232/prepub

## Supplementary Material

Additional file 1 **Appendix 1.** Incidence of and risk factors for reporting an ILI using the fever definition. **Appendix 2.** Risk factors of having at least one ILI using fever definition. **Appendix 3.** Risk factors of having at least one ILI with children removed. Click here for file

## References

[B1] WHO Influenza (seasonal) Fact sheet N°211 http://www.who.int/mediacentre/factsheets/fs211/en/index.html

[B2] Baguelin M Hoschler K Stanford E Waight P Hardelid P Andrews N Miller E Age-Specific Incidence of A/H1N1 2009 Influenza Infection in England from Sequential Antibody Prevalence Data Using Likelihood-Based Estimation PLoS One 2011 14 2 e17074 10.1371/journal.pone.0017074 21373639 PMC3044152

[B3] Elliot A Powers C Thornton A Obi C Hill C Simms I Waight P Maguire H Ford D Povey E Monitoring the emergence of community transmission of influenza A/H1N1 in England: a cross sectional opportunistic survey of self sampled telephone callers to NHS Direct Br Med J 2009 14 2153 2157 10.1136/bmj.b3403 PMC2733951 19713236

[B4] Brooks-Pollock E Tilston N Edmunds WJ Eames KT Using an online survey of healthcare-seeking behaviour to estimate the magnitude and severity of the 2009 H1N1v influenza epidemic in England BMC Infect Dis 2011 14 68 10.1186/1471-2334-11-68 21410965 PMC3073914

[B5] Friesma I Kopperschaar C Donker G Dijkstra F van Noort S Smallenburg R van der Hoek W van der Sande M Internet-based monitoring of influenza-like illness in the general population: experience of five influenza seasons in The Netherlands Vaccine 2009 14 45 6353 10.1016/j.vaccine.2009.05.042 19840672

[B6] Vandendijc Y Faes C Hens N Eight years of the great influenza survey to monitor influenza-like illness in Flanders PLoS One 2013 14 5 e64156 10.1371/journal.pone.0064156 23691162 PMC3656949

[B7] Eames KT Tilston NL Brooks-Pollock E Edmunds WJ Measured dynamic social contact patterns explain the spread of H1N1v influenza PLoS Comput Biol 2012 14 3 e1002425 10.1371/journal.pcbi.1002425 22412366 PMC3297563

[B8] Carlson SJ Dalton CB Durrheim DN Fejsa J Online Flutracking Survey of Influenza-like Illness during Pandemic (H1N1) 2009, Australia Emerg Infect Dis 2010 14 12 1960 1962 10.3201/eid1612.100935 21122231 PMC3294562

[B9] You FN Flu Near You [10/07/2013] Available from: https://flunearyou.org/

[B10] Eames KT Brooks-Pollock D Paolotti D Perosa M Gionannini C Edmunds WJ Rapid assessment of influenza vaccine effectiveness: analysis of an internet-based cohort Epidemiol Infect 2012 14 7 1309 1315 10.1017/S0950268811001804 21906412

[B11] Tilston NL Eames KT Paolotti D Ealden T Edmunds WJ Internet-based surveillance of Influenza-like-illness in the UK during the 2009 H1N1 influenza pandemic BMC Public Health 2010 14 650 10.1186/1471-2458-10-650 20979640 PMC2988734

[B12] Office for National Statistics Datasets and reference tables Available from: http://www.ons.gov.uk/ons/datasets-and-tables/index.html?pageSize=50&sortBy=none&sortDirection=none&newquery=population+structure&content-type=Reference+table&content-type=Dataset

[B13] HPA HPA Weekly National Influenza Report 2013 [cited 2013 25/03/2013] Available from: http://www.hpa.org.uk/webc/hpawebfile/hpaweb_C/1317138449850

[B14] England PH Surveillance of influenza and other respiratory viruses, including novel respiratory viruses, in the United Kingdom: Winter 2012/13 Available from: http://www.hpa.org.uk/webc/HPAwebFile/HPAweb_C/1317139321787

[B15] Biggerstaff M Jhung MA Reed C Garg S Balluz L Fry AM Finelli L Impact of medical and behavioural factors on influenza-like illness, healthcare-seeking, and antiviral treatment during the 2009 H1N1 pandemic: USA, 2009-2010 Epidemiol Infect 2013 14 1 114 125 23522400 10.1017/S0950268813000654 PMC4608246

[B16] McCaw JM Howard PF Richmond PC Nissen M Sloots T Lambert SB Lai M Greenberg M Nolan T McVernon J Household transmission of respiratory viruses – assessment of viral, individual and household characteristics in a population study of healthy Australian adults BMC Infect Dis 2012 14 345 10.1186/1471-2334-12-345 23231698 PMC3538067

[B17] Klein S Hodgson A Robinson D Mechanisms of sex disparities in influenza pathogenesis J Leukoc Biol 2012 14 1 67 73 10.1189/jlb.0811427 22131346 PMC4046247

[B18] Biggerstaff M Jhung M Kamimoto L Balluz L Finelli L Self-reported influenza-like illness and receipt of influenza antiviral drugs during the 2009 pandemic, United States, 2009-2010 Am J Public Health 2012 14 10 e21 e26 10.2105/AJPH.2012.300651 22897525 PMC3490664

[B19] Heymann AD Hoch I Valinsky L Kokia E Steinberg DM School closure may be effective in reducing transmission of respiratory viruses in the community Epidemiol Infect 2009 14 10 1369 1376 10.1017/S0950268809002556 19351434

[B20] Cauchemez S Valleron AJ Boelle PY Flahault A Ferguson NM Estimating the impact of school closure on influenza transmission from Sentinel data Nature 2008 14 7188 750 754 10.1038/nature06732 18401408

[B21] Eames KT Tilston NL Edmunds WJ The impact of school holidays on the social mixing patterns of school children Epidemics 2011 14 2 103 108 10.1016/j.epidem.2011.03.003 21624781

[B22] Castilla J Godoy P Dominguez A Martin V Delgado-Rodriguez M Martinez-Baz I Baricot M Soldevila N Mayoral JM Astray J Quintana JM Canton R Castro A Gonzalez-Candelas F Alonso J Saez M Tamames S Pumarola T Risk factors and effectiveness of preventive measures against influenza in the community Influenza Other Respir Viruses 2013 14 2 177 183 10.1111/j.1750-2659.2012.00361.x 22458533 PMC5780759

[B23] Troko J Myles P Gibson J Hashim A Enstone J Kingdon S Packham C Amin S Hayward A Nguyen Van-Tam J Is public transport a risk factor for acute respiratory infection? BMC Infect Dis 2011 14 16 10.1186/1471-2334-11-16 21235795 PMC3030548

[B24] Kamimoto L Euler GL Lu PJ Reingold A Hadler J Gershman K Farley M Terebuh P Ryan P Lynfield R Albanese B Thomas A Craig AS Schaffner W Finelli L Bresee J Singleton JA Seasonal influenza morbidity estimates obtained from telephone surveys, 2007 Am J Public Health 2013 14 4 755 763 10.2105/AJPH.2012.300799 23237164 PMC3673269

