# Supplementary material for: Incidence and risk factors for influenza-like-illness in the UK: online surveillance using Flusurvey
Source: BMC Infect Dis. 2014 May 1;14:232. doi: 10.1186/1471-2334-14-232 (PMC4025540; doi:10.1186/1471-2334-14-232)
Supplement: Additional file 1 — Appendix 1. Incidence of and risk factors for reporting an ILI using the fever definition. Appendix 2. Risk factors of having at least one ILI using fever definition. Appendix 3. Risk factors of having at least one ILI with children removed. [file 1471-2334-14-232-S1.docx]

Appendix 1. Incidence of and risk factors for reporting an ILI using the fever definition

|  | ILI Fever definition | |
| --- | --- | --- |
|  | Overall incidence (at least once) | Incidence per report |
| 0-17 | 40.1 | 5.2 |
| 18-24 | 25.2 | 3.4 |
| 25-34 | 32.9 | 3.9 |
| 35-44 | 42.1 | 5.1 |
| 45-64 | 39.8 | 4.0 |
| 65+ | 16.4 | 1.2 |
| Male | 28.8 | 2.8 |
| Female | 39.2 | 4.3 |
| East Midlands | 38.1 | 3.6 |
| East England | 32.8 | 3.5 |
| London | 30.2 | 3.1 |
| North East | 36.3 | 4.0 |
| North West | 39.6 | 4.3 |
| Northern Ireland | 53.1 | 6.9 |
| Scotland | 38.0 | 3.8 |
| South Central | 33.0 | 3.6 |
| South East Coast | 38.2 | 4.2 |
| South West | 39.1 | 4.3 |
| Wales | 34.3 | 3.5 |
| West Midlands | 35.5 | 3.2 |
| Yorkshire & Humberside | 38.4 | 4.3 |

Appendix 2: Risk factors of having at least one ILI using fever definition

| Variable | OR | 95% CI | | P |
| --- | --- | --- | --- | --- |
| Female | 1.43 | 1.25 | 1.65 | <0.001 |
| Unvaccinated | 2.27 | 1.92 | 2.70 | <0.001 |
| 18-24 | 0.58 | 0.38 | 0.89 | 0.012 |
| 25-34 | 0.99 | 0.71 | 1.38 | 0.941 |
| 35-44 | 1.52 | 1.11 | 2.08 | 0.008 |
| 45-64 | 1.55 | 1.14 | 2.10 | 0.005 |
| 65+ | 0.76 | 0.50 | 1.13 | 0.178 |
| Contact with children | 1.51 | 1.26 | 1.81 | <0.001 |
| Lives with Children | 1.11 | 0.96 | 1.30 | 0.167 |
| Smoker | 1.33 | 1.07 | 1.65 | 0.010 |
| Take public transport | 0.84 | 0.72 | 0.99 | 0.033 |
| Underlying health condition | 1.38 | 1.14 | 1.68 | <0.001 |
| Employment status | 0.91 | 0.76 | 1.08 | 0.276 |

Appendix 3: Risk factors of having at least one ILI with children removed

| Variable | OR | 95% CI | | P |
| --- | --- | --- | --- | --- |
| Female | 1.56 | 1.36 | 1.79 | <0.001 |
| Unvaccinated | 2.00 | 1.71 | 2.35 | <0.001 |
| 25-34 | 1.19 | 0.86 | 1.65 | 0.291 |
| 35-44 | 1.52 | 1.09 | 2.10 | 0.012 |
| 45-64 | 1.62 | 1.19 | 2.21 | 0.002 |
| 65+ | 1.04 | 0.71 | 1.52 | 0.851 |
| Contact with children | 1.51 | 1.25 | 1.84 | <0.001 |
| Lives with Children | 1.11 | 0.96 | 1.30 | 0.170 |
| Smoker | 1.33 | 1.07 | 1.66 | 0.010 |
| Take public transport | 0.91 | 0.78 | 1.06 | 0.236 |
| Underlying health condition | 1.55 | 1.29 | 1.87 | <0.001 |
| Employment status | 0.93 | 0.78 | 1.11 | 0.425 |
